# Objective risk stratification of prostate cancer using machine learning and radiomics applied to multiparametric magnetic resonance images

**DOI:** 10.1038/s41598-018-38381-x

**Published:** 2019-02-07

**Authors:** Bino Varghese, Frank Chen, Darryl Hwang, Suzanne L Palmer, Andre Luis De Castro Abreu, Osamu Ukimura, Monish Aron, Manju Aron, Inderbir Gill, Vinay Duddalwar, Gaurav Pandey

**Affiliations:** 1 Department of Radiology, University of Southern California, Los Angeles, CA USA; 2 USC Institute of Urology, Los Angeles, CA USA; 3 Department of Pathology, University of Southern California, Los Angeles, CA USA; 4 Department of Genetics and Genomic Sciences and Icahn Institute for Genomics and Multiscale Biology, Icahn School of Medicine at Mount Sinai, New York, NY USA

## Abstract

Multiparametric magnetic resonance imaging (mpMRI) has become increasingly important for the clinical assessment of prostate cancer (PCa), but its interpretation is generally variable due to its relatively subjective nature. Radiomics and classification methods have shown potential for improving the accuracy and objectivity of mpMRI-based PCa assessment. However, these studies are limited to a small number of classification methods, evaluation using the AUC score only, and a non-rigorous assessment of all possible combinations of radiomics and classification methods. This paper presents a systematic and rigorous framework comprised of classification, cross-validation and statistical analyses that was developed to identify the best performing classifier for PCa risk stratification based on mpMRI-derived radiomic features derived from a sizeable cohort. This classifier performed well in an independent validation set, including performing better than PI-RADS v2 in some aspects, indicating the value of objectively interpreting mpMRI images using radiomics and classification methods for PCa risk assessment.

## Introduction

Prostate cancer (PCa) is the third most common cause of death and the most prevalent male malignancy worldwide^1^. In 2018, the American Cancer Society (ACS) estimates 164,690 new PCa cases (9.5% of all new cancer cases) and 29,430 PCa-related deaths in the United States, imposing a substantial socioeconomic burden. The ability to accurately assess the aggressiveness risk of a diagnosed PCa could improve the selection of appropriate treatment for these patients, leading to improved outcomes, including PCa-specific mortality^2^.

Over the past decade, multi-parametric magnetic resonance imaging (mpMRI) has become increasingly important for the evaluation, localization, and staging of PCa^3,4^. In combination with the Prostate Imaging Reporting and Data System Version 2 (PI-RADS v2), encouraging results have been reported for the prediction of the likelihood of intermediate- and high-grade cancers^5,6^. However, despite the high sensitivity of mpMRI, the assessment of PCa is based on visual qualification and is therefore subjective^6^. The reported inter-observer agreement has only been moderate to good^5,6^, with several multi-reader studies finding an overall inter-reader agreement ranging from poor (0.5) to reasonable (0.71), depending on the study and reader experience^5–8^.

To make mpMRI imaging more objective and reliable, researchers have attempted to identify quantitative imaging parameters extracted from T2-weighted (T2W) and diffusion-weighted (DW) images. T2W and DW (including apparent diffusion coefficient [ADC] map) signal intensities have been shown to correlate with histopathology-based nuclear cell-density^9^, as well as PCa aggressiveness^10,11^. T2W and DW/ADC signal intensities have also been found to correlate with Gleason scores, a histologic scale of PCa dysplasia (abnormal organization of cells)^12,13^; however, the ranges of ADC values for different Gleason scores overlapped considerably, limiting their utility in differentiating between these scores, and clinical decision-making^11,12^.

The field of radiomics deals with the extraction of quantifiable features such as texture, size and shape from clinical images^14–16^. The underlying assumption is that images collected during routine clinical care contain latent information regarding tumor behavior that can be extracted using a variety of quantitative image characterization algorithms^14^. The extraction of these radiomic features enables the conversion of collections of digital clinical images into structured quantitative data that can help model tumor behavior. For example, in radiomic studies conducted using mpMRI, a variety of tumor phenotypes relating to texture aided in the diagnosis of PCa^17–19^. Using five different Haralick texture features^20^ (entropy, energy, correlation, inertia, and homogeneity) extracted from T2W and DW images, Wibmer *et al*. showed significant differences in all the features between cancerous and non-cancerous tissue in the peripheral zone^19^. Lv *et al*. used fractal-based features to distinguish prostate tumor tissue from normal peripheral zone tissue^18^.

Machine Learning (ML) methods are designed to sift through large amounts of high-dimensional data, without any particular guiding (biomedical) hypothesis, to directly discover potentially actionable knowledge^21,22^. Due to these abilities, ML methods, especially those for classification^21,22^, are increasingly being incorporated into radiomic studies to improve PCa^23,24^ assessment and make it less subjective. A probabilistic Support Vector Machine (SVM)^25^ applied to a combination of Haralick features^20^ was found to perform well in discriminating benign lesions from malignant PCa^26^. For the same problem, Liu *et al*. reported an AUC^27^ of 0.73 based on mpMRI-based radiomic features and SVM, which increased to 0.82 when combined with targeted prostate biopsy results, although the results were based on a small 18 patient validation set^28^. Tiwari *et al*. used a similar approach for distinguishing benign lesions from PCa regions, as well as high- from low-grade PCa^29^.

Although these and other studies^23,24^ have demonstrated the utility of combining radiomics and ML for PCa assessment, they have only explored this combination in a limited manner. These limitations include the utilization of a small number of classification methods, often of the same type (e.g. SVM), evaluation using the AUC score only, and a non-rigorous assessment of all possible combinations of radiomics and classification methods to identify the best possible classifier. This study presents a systematic and rigorous ML-based framework comprised of classification^30^, cross-validation^31^ and statistical analyses^32^ designed to identify the best performing classifier for PCa risk stratification based on mpMRI-derived radiomic features derived from a sizeable cohort. Note that risk stratification of PCa using NCCN guidelines^33^ is a more challenging task compared to other PCa outcomes predicted in the studies discussed above, such as pathologic grade, as it includes an interplay of multiple factors. To conduct a comprehensive assessment of the candidate classifiers tested in this framework, the Precision-Recall-F-measure family of evaluation measures was employed in addition to the AUC score^27^. This family is more informative about classifier performance in scenarios of unbalanced class distributions^34,35^, typical in biomedical studies like PCa risk stratification, as is also the case in this and other studies’ cohorts^36,37^. The performance of the final classifier developed by the framework for assessing risk was evaluated in an independent cohort of PCa patients, and compared to the PI-RADS v2 system^5^ to assess the relative utility of a well-developed combination of radiomics and ML for objective and accurate PCa risk stratification.

## Results

In this section, we present the results of the application of our ML-based framework and its components on radiomics features derived from our various study cohorts.

### Impact of random over-sampling on classification performance

To address the substantial class imbalance in the development set, i.e., a much higher number of lower-risk patients (54) than high-risk ones (14), the performance of the classification algorithms constituting the framework was first evaluated with and without random over-sampling. For all the algorithms, random over-sampling led to significantly improved performance across all the evaluation measures as compared to no oversampling (Wilcoxon signed rank-sum test p < 0.02; Supplementary Fig. 1). Thus, only results based on random over-sampling are presented below.

### Classifier evaluation in our framework

**Figure 1:**
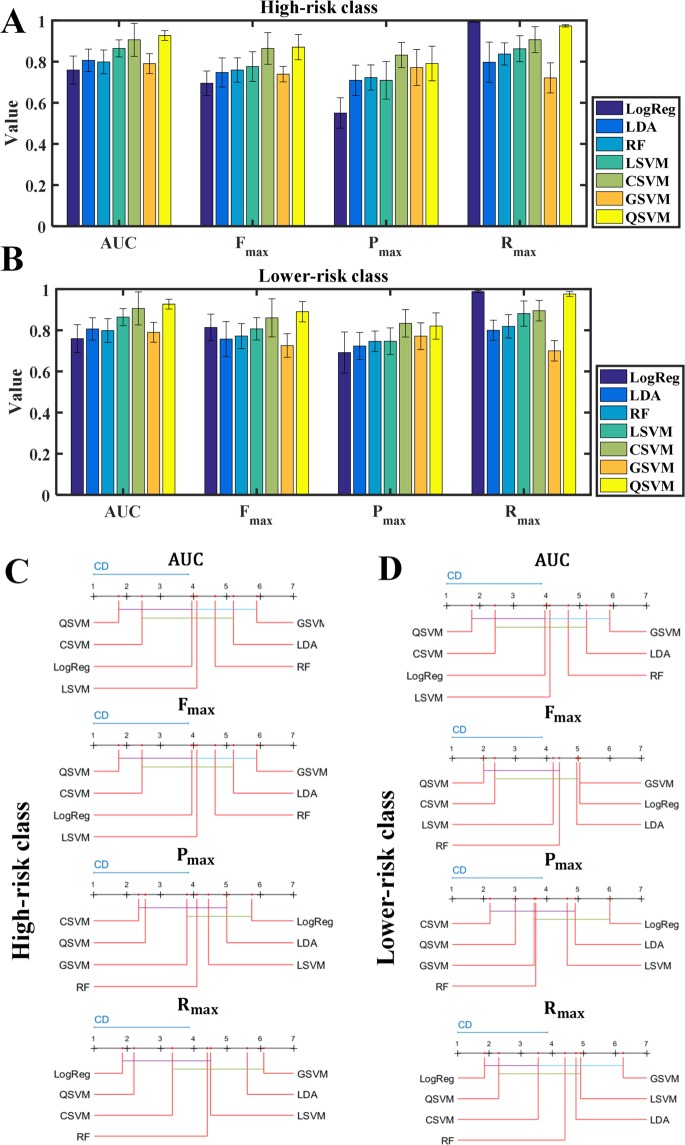
Results of the performance evaluation of various classification algorithms and their resultant models tested in our framework, grouped by several evaluation measures (**A**): High-risk class; (**B**): Lower-risk class). Also shown are the results of the statistical comparison of these performances in the form of Critical Difference (CD) plots for the high (**C**) and lower (**D**) PCa risk classes respectively. Classification algorithms, represented by vertical + horizontal lines, are displayed from left to right in terms of the average rank obtained by their resultant models in each of the ten cross-validation rounds, and the classifiers producing statistically equivalent performance are connected by horizontal lines. These results show that the Quadratic kernel-based SVM (QSVM) is the best performer overall, especially because it is the only classifier that is statistically the best performer (leftmost classifier in the plots, either by itself or tied with another classifier like CSVM or LogReg) in terms of all the evaluation measures for both the classes. The CD plots were drawn using open-source Matlab code.

The results of the various classification algorithms and models evaluated in a cross-validation setting in the ML framework are shown in Fig. 1. The Quadratic kernel-based SVM (QSVM)^25^ classifier achieved the highest overall absolute performance in terms of the average AUC (0.92) across the ten cross-validation runs. In terms class-specific measures also, QSVM attained the highest average values of F_max_ (0.89), P_max_ (0.82) and R_max_ (0.98) for the high-risk class (Fig. 1A) and F_max_ (0.87), P_max_ (0.79) and R_max_ (0.97) for the lower-risk class (Fig. 1B).

Since absolute performance measures can sometimes be misleading, a comprehensive statistical comparison of the performance of the classification algorithms tested in the framework was also conducted using Friedman-Nemenyi tests^32^. The visualization of the results as Critical Difference (CD) plots^32^ (Fig. 1C,D) show that the QSVM^25^ is statistically the best performer (leftmost classifier in the plots, either by itself or tied with another classifier like CSVM^25^ or LogReg^38^) in terms of all the evaluation measures for both the classes. Thus, QSVM was chosen as the algorithm to learn the final radiomics-based classifier over the whole development set. The associated threshold for binarizing the probabilistic predictions generated by the classifier into discrete high/lower-risk labels was determined to be 0.17253, the average of the thresholds found to maximize F_max_ for QSVM in the ten cross-validation runs.

### Evaluation of final classifier on independent validation set

To assess the generalizability of the performance of the final classifier, it was applied to an independent validation cohort of 54 PCa patients and its performance was compared to that of PI-RADS v2 in terms of AUC, F-measure, Precision and Recall (Table 1). Although the classifier performed equivalently with PI-RADS v.2 in terms of AUC (considering the standard errors), it performed substantially better in terms of the class-specific measures (F-measure, Precision and Recall), especially for the high-risk class. P-values of the bootstrapping and Friedman-Nemenyi test-based performance comparison procedure (third row of Table 1) showed that the classifier performed equivalently with PI-RADS in terms of AUC, but performed significantly better (p << 0.05) than in terms of the class-specific measures. This is because the classifier, which is strengthened for identifying high-risk samples through random oversampling, is able to more accurately and quantitatively classify the patients into the correct risk classes, as compared to the generally qualitative PI-RADS v2^39^. The radiomics classifier also performed much better on the real validation set than its randomized versions (Table 1), indicating that the classifier did capture a *real* relationship between the radiomics features and PCa risk status.

**Table 1:** Evaluation of the final QSVM-based radiomics classifier and alternatives/benchmarks on the independent validation set of 53 PCa patients in terms of several performance measures.

Classifier/Benchmark	AUC	High-risk (minority) class			Lower-risk (majority) class		
		F-measure	Precision	Recall	F-measure	Precision	Recall
PI-RADS v2	0.73	0.52	0.45	0.61	0.82	0.87	0.78
QSVM-based radiomics classifier	0.71 (0.673)	0.69 (4.37 × 10^−30^)	0.57 (2.07 × 10^−26^)	0.86 (2.00 × 10^−25^)	0.85 (4.12 × 10^−16^)	0.94 (2.90 × 10^−19^)	0.78 (5.58 × 10^−6^)
Randomized validation sets	0.51 (0.01)	0.45 (0.02)	0.35 (0.03)	0.62 (0.02)	0.69 (0.02)	0.82 (0.02)	0.6 (0.03)

The p-values obtained from the Friedman-Nemenyi test-based comparison of the performance of PI-RADS and radiomics classifier in terms of all the evaluation measures are shown in parentheses in the fourth row. Standard errors for the measures on the 100 randomized validation sets are shown in parentheses in the fifth row. The radiomics classifier produces more accurate predictions than PI-RADS v2, especially in terms of class-specific measures (F-measure, Precision and Recall) that are more meaningful for unbalance class situations like the cohorts in our study. It also performed better on the real validation set than its randomized versions, indicating that the classifier did capture a *real* relationship between the radiomics features and PCa risk status.

## Discussion

Identifying patients with clinically significant PCa remains a challenging problem^40^. Although mpMRI is a useful tool for this purpose, the reported inter-observer agreement among its interpretations has been variable, with scores ranging from poor (0.5) to reasonable (0.71), depending on the study and reader experience^5–8^. This variation has been attributed to experience of the imaging and pathology readers, variable interpretation of PI-RADS v2 guidelines and diversity of standards used for histopathological staging^41^.

Radiomics can analyze a large number of features of images that are difficult to study solely by visual assessment^42^. Furthermore, radiomics, in combination with machine learning (ML), can help achieve objective classification of clinical images that can be a valuable tool to aid clinicians in identifying appropriate treatment options for patients without subjecting them to unnecessary interventions^4^. In contrast to prior studies following the radiomics followed by ML methods approach^23,24,26,28,29^, which generally utilized only a single or small number of ML methods, this study developed a systematic and rigorous ML-based framework for deriving a reliable and objective risk classifier by finding the optimal classification method(s) for radiomic features derived a given set of imaging data. This framework was used to develop a radiomics-based classifier operating on mpMRI images of PCa tumors that accurately distinguishes between subjects with high-risk PCa and those with lower-risk PCa. Our classifier, built on top of 110 radiomic features interpreted via a Quadratic kernel-based Support Vector Machine (QSVM)^25^, performed with reasonably high precision or predictive value (PPV = 0.57 and NPV = 0.94) and high recall or sensitivity (0.86 and 0.72 for the high and lower-risk classes respectively) for classifying PCa patients in an independent validation set (Table 1). This effectiveness of QSVM can be partly attributed to the use of kernels (square of the dot product in this case) that can represent non-Euclidean similarity or distance between data points without having to transform the points into the non-Euclidean space^25^. This classifier performed better than PI-RADS v2 on the same validation set, especially in terms of the class-specific evaluation measures (precision, recall and F-measure)^27^, indicating the value of objective assessment of PCa risk as compared to a more subjective one.

The Area Under the ROC Curve (AUC) is routinely used to assess the performance of classification models^27^, including in radiomics studies^43–47^. However, AUC weighs classification errors in the two classes being evaluated equally in a cumulative manner, which can lead to misleading results in situations where the study data may have substantially imbalanced numbers of samples in the two classes^34,35^. Since this study’s cohorts were similarly imbalanced, i.e. many more lower-risk patients as compared high-risk ones, the performance of all the classifiers tested in the framework, both candidate and final, was also assessed in terms of class-specific measures, namely recall (sensitivity), precision (predictive value), and F-measure (Supplementary Fig. 2), in addition to AUC. These measures enabled us to specifically evaluate the classifiers’ performance on the minority class in this study’s cohorts, namely high-risk PCa, which can be dominated by the majority class during classifier testing and evaluation^48^, a phenomenon that may not be adequately revealed by AUC.

The design of our single-center, retrospective study has some limitations. First, the study only used data from a single type of scanner from a single MRI vendor, obtained using the same imaging protocol. This was done to keep the radiomics features as comparable as possible across patients. It is well-recognized that radiomic features need to be reliable, i.e., reproducible and repeatable across different imaging and image-processing protocols, as well as scanners. However, currently, a lot of the work assessing reliability has only been reported on CT-based radiomic features^49–53^, and the work is relatively limited for MRI-based radiomic features. Mayerhoefer *et al*. performed basic investigations using simple polystyrene/agar gel-based phantoms to evaluate the effect of MRI acquisition parameters such as repetition time, echo time, number of acquisitions and sampling bandwidth on image texture, an important radiomic feature^54^. The study summarized significant changes in this feature with changes in the tested MRI acquisition parameters. Collewet *et al*. drew similar conclusions during their investigation of the reliability of texture-based classification of a small number of old and new cheese samples^55^. In summary owing to the increased number of imaging variables within MRI compared to CT, as well as the lack of reliable phantoms (imaging standards) and standardized reliability tests, it was beyond the scope of our study to assess the reliability of the radiomics features used. Further studies are warranted on this topic.

Also, due to our stringent approach to acquire controlled data, our sample size, while quite moderate, was comparable to similar exploratory studies^24,42,56^. Furthermore, the number of samples in the development set in our study (n = 68) was comparable to (≈) the number of features (p = 110), similar to exploratory studies^24,42,56^ using MRI data. Although, some radiomics studies use feature selection techniques to reduce the number of features^57^, the n ≈ p nature of our study doesn’t necessitate the use of these techniques^58^. Indeed, with larger values of n and p, this step may become much more important and useful, as has been demonstrated in other studies^59,60^.

Another potential challenge for our study is that it is based on a relatively imbalanced cohort, but this is still comparable to those used in prior similar studies^36^. We addressed the imbalance problem by using the commonly used method of randomly oversampling the minority class^48^ (high-risk PCa) during classifier training, which yielded significant performance improvements (Supplementary Fig. 1). The potential overfitting of the classifier due to the relatively small size of our cohort was also addressed by adopting a rigorous cross-validation setup combined with statistical analyses of classifier performance in our framework. The performance of the QSVM-based final classifier was also validated on an independent patient cohort, whose results gave us confidence that the classifier is not over-fit to the data, especially because it performed better than PI-RADS v2.

While PI-RADS is more easy-to-do and practical than running machine learning from a scratch, the technique is based on qualitative evaluation and therefore subjective in nature^5,6^. The goal of incorporating machine learning into radiomics is not to compete with the radiologist, but to rather provide the radiologist and physician team taking care of the patient with objective prediction tools that can aid personalized decision making regarding individual disease course and treatment outcome^61,62^. The use of such decision support systems will enhance the quality of the radiologist’s work and help in the long-run aid in the integration of such systems into routine patient care^61^. We would also like to emphasize that, although the machine learning-based classifier derivation process may seem involved, the clinical practitioners don’t have to deal with it directly. The resultant classifier, which can typically be implemented in a few lines of code on top of the existing radiomics pipeline, can be executed by such practitioners through a simple GUI and only a few clicks of a mouse or key. This study can be expanded and improved upon by employing larger cohorts for developing and validating the risk stratifier, testing classification algorithms beyond those available in Matlab’s classification-learner package and qualitatively interpreting the stratifier to inform radiomics, clinical imaging and possibly even PCa biology. Our present and future efforts are expected to improve (PCa) patient stratification that can eventually translate to more effective and personalized patient treatments, wherein decisions regarding active surveillance or intensified therapy can be made more objectively and reliably.

## Materials and Methods

### Study Cohorts

Seventy three prostate cancer (PCa) patients presenting between March 2013 and May 2016 were included in this single institution, retrospective study. Inclusion criteria were: (1) histopathologic diagnosis of PCa, (2) mpMRI of the prostate, and (3) transrectal ultrasound-magnetic resonance (TRUS-MR) imaging fusion guided biopsy of the prostate within 2 months of mpMRI. Five patients were excluded from the original set due to marked imaging artifacts from hip prostheses, resulting in the final development set consisting of 68 patients. For patients with multiple lesions identified on mpMRI, the dominant lesion was chosen. The patients were divided into high, intermediate and low categories per National Comprehensive Cancer Network guidelines^33^. These categories were subsequently combined into 2 classes - “high risk” (high risk and above as per the above guidelines) and “lower risk” (intermediate and low risk as per the above guidelines) - to make the data amenable for traditional classification algorithms.

The development set was used to train and evaluate all the candidate classifier for differentiating between the above classes, as well as for training the final classifier. This classifier was applied to an independent validation set of 53 PCa patients who presented at the USC Keck Medical Center between June 2016 and July 2017 after applying the same inclusion and exclusion criteria used for constructing the development set. Baseline characteristics of the development and validation sets are provided in Table 2. The study was approved by the USC institutional review board, and all methods were performed in accordance with the relevant guidelines and regulations.

**Table 2:** Baseline characteristics of the patients and their PCa tumors in the development and test sets.

	Development Set (N = 68)			Validation Set (N = 53)			Development vs. Validation set t-test p-value
	All	Lower risk (N = 54; 79.40%)	High risk (N = 14; 20.60%)	All	Lower risk (N = 39; 73.58%)	High risk (N = 14; 26.41%)	
Gleason	6.93 (0.86)	6.58 (0.55)	8.00 (0.65)	7.13 (0.91)	6.43 (0.07)	8.36 (0.71)	0.21
PSA	7.51 (4.54)	5.99 (2.31)	12.46 (5.99)	8.26 (9.42)	5.33 (2.73)	15.55 (5.45)	0.60
PI-RADS v2	3.85 (0.60)	3.71 (0.51)	4.21 (0.56)	3.79 (0.74)	3.31 (0.48)	4.36 (0.81)	0.63

In addition to the mean value of each characteristic, its standard deviation is shown in parentheses. The p-values from the t-test of the comparison of the characteristics between the development and validation sets, shown in the final column, demonstrate that there are no significant differences between the two sets that could potentially bias the classification results.

### Imaging and Radiomics

#### Image acquisition

MRI examinations were performed on a 3-Tesla MRI system (GE Healthcare, WI, Milwaukee) using a standard protocol compliant with PI-RADS v2^5^. Images containing the largest lesion diameter in transaxial, coronal and sagittal planes were saved for radiomics analysis.

#### mpMRI image segmentation

The acquired images were transferred to a dedicated Synapse 3D workstation (Fujifilm Medical Systems U.S.A., Stamford, CT). A radiologist experienced in prostate imaging identified regions of interest (ROI), and an ROI was manually placed within the margins of the tumor in a two-dimensional (2D) plane. The mpMRI images were also qualitatively evaluated by the same radiologist using PI-RADS v2^5,6^ who was blinded to all clinic-pathological information about the patients to avoid bias.

#### Radiomic feature extraction

**Figure 2:**
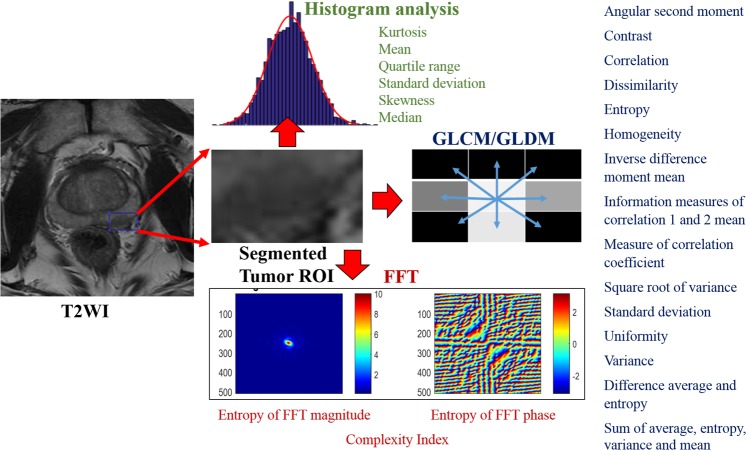
Flowchart of some sample quantitative radiomic features used in our study that were extracted from segmented tumor regions of interest (ROI) of mpMRI images. In summary, 55 different features were extracted per image type (i.e., T2WI or ADC) using four different texture extraction methods, yielding 110 radiomic features per patient. The four texture methods included histogram analysis, Gray-Level Co-occurrence and Difference Matrix methods (GLCM and GLDM) and Fast Fourier Transform (FFT). Some of these features are highlighted in green, blue and red respectively. The full list and details of these features are provided in the online Appendix in Supplementary Information. Note that all these features were 2D, as the input imaging data were two-dimensional.

2D radiomic analysis was conducted on the orientation that provided the largest tumor diameter in each phase in the axial, coronal or sagittal dimension of both the T2W and ADC images. Fifty-five different texture features each were derived from both T2W and ADC images using four different methods, namely histogram analysis, Gray-Level Co-occurrence and Difference Matrices (GLCM and GLDM*)*, and Fast Fourier transform (FFT)-based frequency features from, totaling 110 radiomic features (Fig. 2; more details in Appendix in Supplementary Information^63^. Note that only 2D radiomic features were used, as the input imaging data were two-dimensional). MATLAB® (Mathworks, Natick, MA) was used to process the voxel-wise data for deriving the features.

### Classification Framework

**Figure 3:**
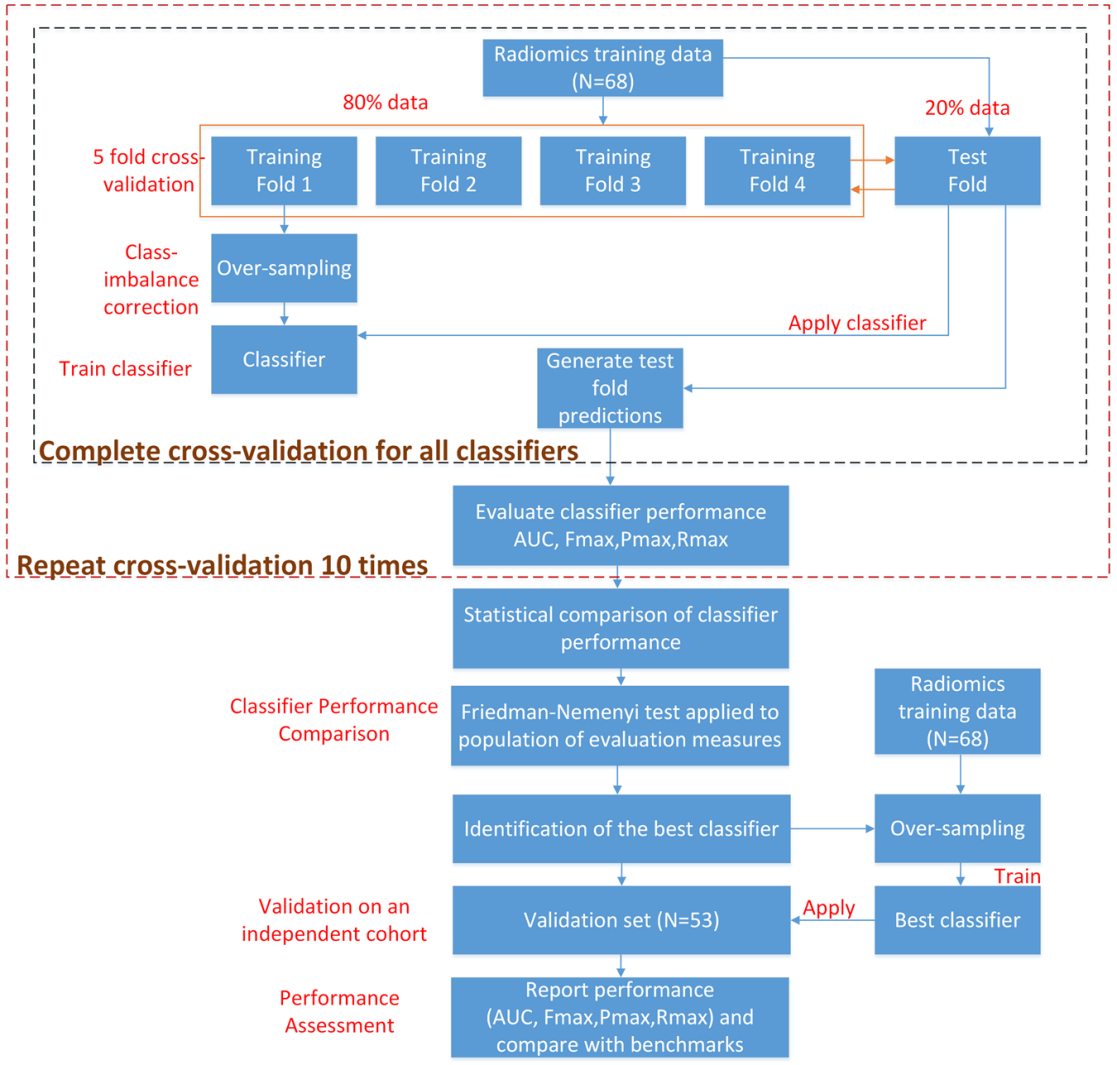
Workflow of our ML-based framework used to identify the best combination of radiomic features and classification algorithm for categorizing PCa patients into high-risk and lower-risk categories. Cross-validation was used to identify the best performing algorithm out of seven commonly used algorithms, which was then used to train the final classifier on the entire development set (68 PCa patients). This classifier was then evaluated on an independent validation set of 53 PCa patients in terms of a variety of performance measures, namely AUC, F_max_, P_max_ and R_max_.

Based on our previous work on developing classifiers from high-dimensional data^59^, we designed a systematic and rigorous ML-based framework comprised of classification^30^, cross-validation^31^ and statistical analyses^32^ to identify the classification model from the development set that most accurately differentiates high-risk PCa patients from lower-risk ones based on mpMRI-derived radiomic features. The main components of the framework, visualized in Fig. 3, are described below:

- *Classification:* Seven classification algorithms, Logistic regression (LogReg)^38^, Linear (L), Quadratic (Q), Cubic (C) and Gaussian (G) kernel-based Support Vector Machine (SVM)^25^, Linear Discriminant Analysis (LDA)^64^ and Random Forest (RF)^65^, were examined for learning candidate classification models from the development set. These algorithms were chosen given their widespread use in classification studies and easy-to-use implementations in the MATLAB® classification-learner package.
- *Random oversampling to address class imbalance:* Many clinical outcomes exhibit an unbalanced natural distribution of patients between different classes, e.g. cancer-afflicted and cancer-free, in a general sampling of the population^66^. This was the case in the development set, where only 14 samples belonged to the high-risk class, as compared to 54 in the lower-risk one. This may create a challenge for traditional classification algorithms, as they are generally designed for (almost) balanced classes^48^. Thus, to address this problem, the random oversampling method^48^, where multiple copies of the minority class samples (here, high-risk PCa patients) in the development set were created to equal the number of the majority class samples (here, lower-risk PCa patients), was used. This resampled development set was then used for training the candidate classifier(s).
- *Cross-validation:* A 5-fold cross-validation (CV) procedure^31^ was applied to the development set to train and evaluate candidate classifiers using the algorithms listed above, supplanted with random oversampling during training. In each CV round, candidate classifiers were trained using 80% of the development set (4 folds), and evaluated on the remaining 20% (1 fold). Repeating the process over all the folds and collecting the resulting predictions generated a vector of the same length as the size of the development set. This vector was then compared with true labels of the patients, using the evaluation measures discussed below, to assess the classification performance of the algorithm being considered. This CV process was repeated ten times to reduce the unlikely chance of getting over-optimistic results with just one run.
- *Statistical analysis of classifier performance:* To determine the best performing classification algorithm among those tested in the 10 CV rounds, a statistical analysis of the performance of all the tested classification models was conducted using the Friedman-Nemenyi tests^32^. These tests, which also account for multiple hypothesis testing, are used to assess the statistical significance of the relative difference of performance of the algorithms and their resultant models in terms of their relative ranks across the 10 CV runs. Critical Difference (CD) plots^32^, implemented using open-source Matlab code^67^, were used to visualize the results of these tests and enabled us to identify the best performing classification algorithm.
- *Final classifier development:* The final classifier was trained by applying the above best performing algorithm to the whole development set.

### Assessment of classifier performance

Classifier performance can be evaluated in terms of a variety of measures^27^. Although the Area under the ROC Curve (AUC) has been the most commonly used performance measures in radiomics studies^68,69^, it is not reliable in cases of substantially unbalanced classes^34,35^, which is the case in this study. Thus, in addition to AUC, class-specific Precision, Recall and F-measure evaluation measures, which are more suited for unbalanced class situations^27,35^ (Supplementary Fig. 2), were also used. Like AUC, these measures range from 0 to 1, with higher values indicating better classification performance. Also, like the ROC curve, a precision-recall curve can be derived by varying the same threshold that is applied to the classification scores and computing these measures, as well as the associated F-measure. The maximum value of F-measure for the high-risk class achieved across all these thresholds, also termed F_max_^70–72^, as well as the associated values of Precision and Recall, termed P_max_ and R_max_ respectively, were used to evaluate the candidate classifiers tested in the framework. The corresponding classification score threshold that yielded this value of F_max_ was also recorded for each of the classifiers. The threshold for the final classifier obtained by averaging the threshold that yielded the highest F_max_ value for the corresponding classification algorithm in each of the ten cross-validation rounds. The final classifier was applied in combination with this threshold to the independent validation set to obtain binary predicted labels for the constituent patients, which were then evaluated in terms of AUC, F-measure, Precision and Recall.

### Validation on an independent patient cohort

The best performing radiomics-based classifier identified by our framework was applied to the independent validation set of 53 PCa patients to assess the classifier’s generalizability to new patient populations. This performance was compared to those of two benchmarks as a part of this assessment:

- *PI-RADS v2:* The images, including T2W and ADC of all ROIs, were scored using PI-RADS v2 by two radiologists with 17 and 4 years of experience in prostate imaging, respectively^5^. The readers were blinded to initial mpMRI reports, clinical data, and pathologic outcomes. Each reader reviewed the MR images, identifying all lesions in each patient suspicious for clinically significant cancer and assigning a score for each lesion based on PI-RADS v2 classification. In scenarios where a discrepancy in score was observed, a consensus was obtained based on a discussion by the two readers. For all patients, the PI-RADS v2 scores following consensus was used for validation tests. A threshold of 3 was used for converting these scores to classify the patients as high and lower risk^33^.
- *Permutation-based randomized validation sets:* To determine the extent to which the performance of our final classifier could have been due to random chance, its performance was also evaluated on 100 randomized versions of the validation set. These versions were obtained by randomly permuting the labels of the samples in the validation set, which is expected to break the relationship between the features and true labels. The final classifier is then applied to each of these randomized versions, and the average of the resultant performance of the classifier was used as the representative evaluation measure of this benchmark.

These performance assessments were carried using the classifier evaluation measures discussed above. To assess the statistical significance of the performance differences between the radiomics classifier and PI-RADS, we created 100 bootstrapped versions of the validation set, and measured the performance of the classifier and PI-RADS on these versions in terms of AUC, recall, precision, and F-measure. Then we evaluated the p-values of the performance differences for each of these measures using the Friedman-Nemenyi tests described above.

### Ethics approval and consent to participate

The institutional review board of the University of Southern California approved the study protocols. Written informed consent was obtained from all subjects and all research was performed in accordance with relevant guidelines and regulations.

## Supplementary information

Supplementary Information
