# Supplementary material for: Objective risk stratification of prostate cancer using machine learning and radiomics applied to multiparametric magnetic resonance images
Source: Sci Rep. 2019 Feb 7;9:1570. doi: 10.1038/s41598-018-38381-x (PMC6367324; doi:10.1038/s41598-018-38381-x)
Supplement: Supplementary file 1 — Supplementary Information [file 41598_2018_38381_MOESM1_ESM.docx]

**Objective risk stratification of prostate cancer using machine learning and radiomics applied to multiparametric magnetic resonance images**

Bino Varghese^a*^, Frank Chen^a^, Darryl Hwang^a^, Suzanne L Palmer^a^, Andre Luis De Castro Abreu^b^, Osamu Ukimura^b^, Monish Aron^b^, Manju Aron^c^, Inderbir Gill^b^, Vinay Duddalwar^a,b^ and Gaurav Pandey^d*^

^a^ *Department of Radiology, University of Southern California, Los Angeles, CA, USA*

^b^ *USC Institute of Urology, Los Angeles, CA, USA*

^c^ *Department of Pathology, University of Southern California, Los Angeles, CA, USA*

^d^ *Department of Genetics and Genomic Sciences and Icahn Institute for Genomics and Multiscale Biology, Icahn School of Medicine at Mount Sinai, New York, NY, USA*

* Corresponding authors: [bino.varghese@med.usc.edu](mailto:bino.varghese@med.usc.edu) and [gaurav.pandey@mssm.edu](mailto:gaurav.pandey@mssm.edu)

**Supplementary Information**


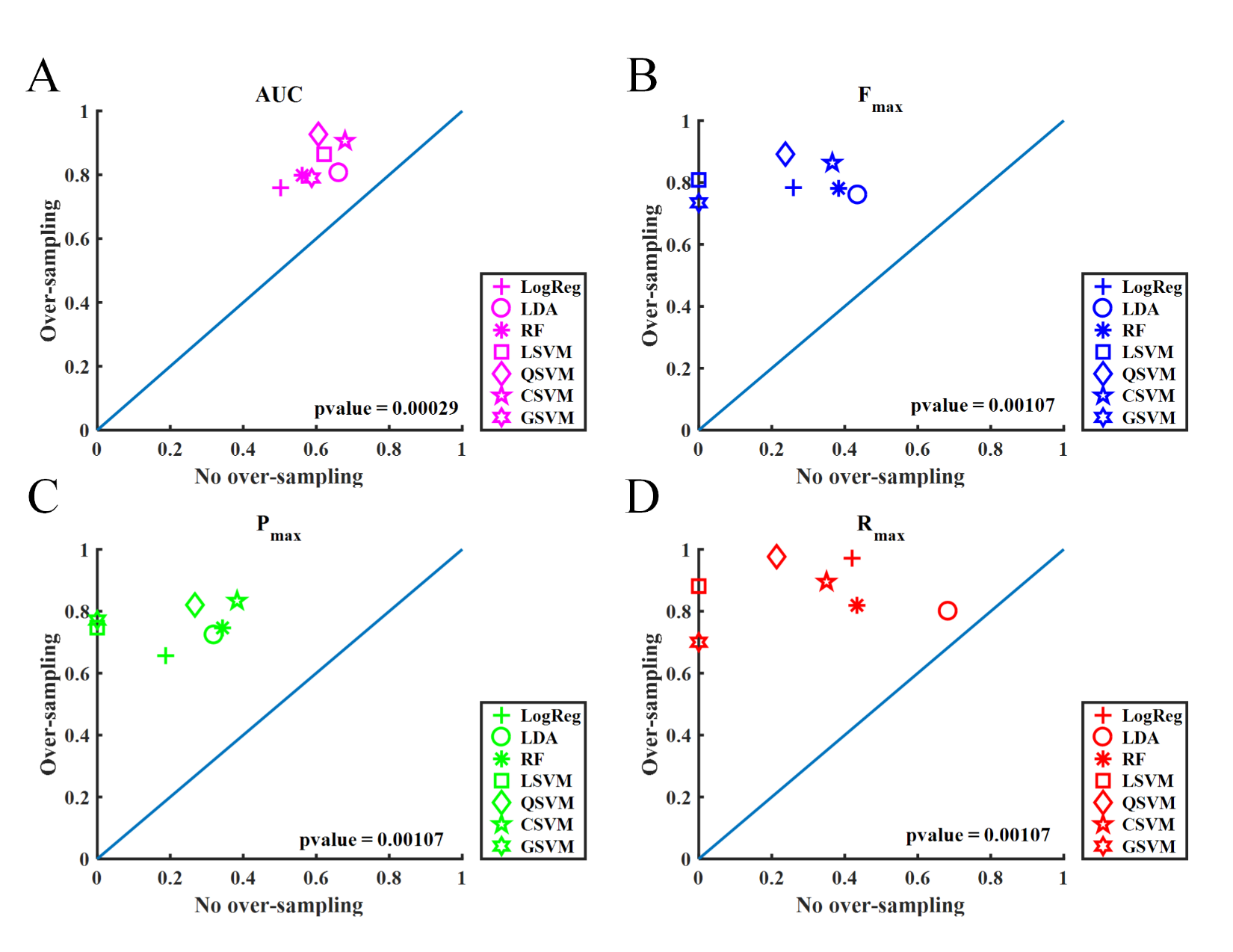


**Supplementary Figure 1:** **Effect of random oversampling (Y-axis) on the cross-validation-derived performance of the various classification algorithms tested, as compared to no oversampling (X-axis), shown in terms of (A) AUC, (B) F_max_, (C) P_max_ and (D) R_max_**. The blue y=x line representing no change is shown for reference. These results show that random oversampling consistently improves classification performance across all algorithms and evaluation measures (Wilcoxon Signed Rank-Sum test p-values for this comparison are shown in the plots).

**
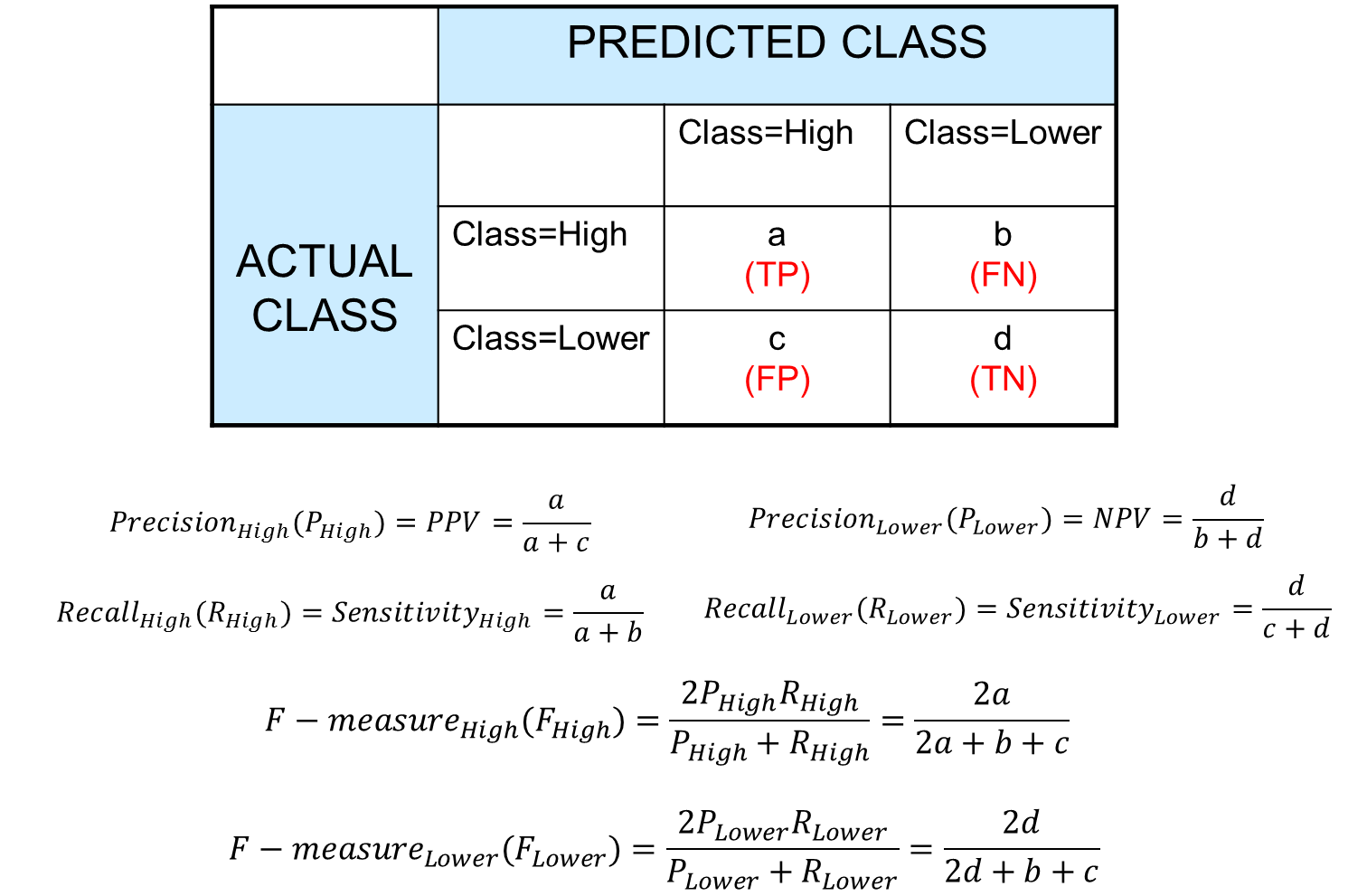
**

**Supplementary Figure 2**: **Evaluation measures for classification models. The relationships between sensitivity, positive/negative predictive values, precision, recall and F-measure are summarized**. F-measure, which is a harmonic (conservative) mean of precision and recall that is computed separately for each class, provides a more comprehensive and reliable assessment of model performance when classes are imbalanced, as is frequently the case in biomedical scenarios.

**Appendix**

Description of the texture-based radiomic features used in the study.

**Definition:**

**Texture (material):** Texture is a measure of the variation of a surface; a rough textured material would have a high rate of change in the high and low points of a surface compared to a smooth textured material.

**Texture (imaging):** If on an image of the surface, the global maximum point was assigned a grayscale value of 65536 (for a 16 bit image), the global minimum point was assigned a grayscale value of 0, and all the points in between had an intermediary value between 0 and 65536, scaled by the ratio of its height in comparison to the global maximum point, image of a rough textured material would have a high rate of change in the high and low points of a surface (grayscale value) compared to a smooth textured material.

**Mathematical descriptions of tumor texture descriptors (here, 55 features)**:

**Imaging based texture descriptors:** Statistical, structural and spectral-based texture assessment are the three main approaches found in literature used to describe texture. Statistical characterization of texture is based on the assessment of texture as measures of statistical properties of the grey levels making up the region of interest. Conventionally, these properties are computed from 1st order statistical methods such as histogram analysis (analysis based on only grayscale values, spatial information is lost), which are easy to implement and understand. Statistical characterization of texture can also be assessed from higher-order texture methods (analysis based on both grayscale values and their spatial orientation) such as Gray-level co-occurrence method (GLCM) and Gray-level difference method (GLDM) Analysis. Structural methods involves techniques of decomposing an image into basic units and identifying the rules required to construct that given image from the basic units. Some examples of structural methods of texture assessment includes fractal analysis. Spectral-based texture features are based on properties of the Fourier spectrum and describe global periodicity of the grey levels of a surface by identifying high-energy peaks in the spectrum and their variations.

Here, we evaluated 4 different types of texture extraction techniques on both the ADC and T2 images. The techniques have been described in literature^1,2^, and are summarized in Figure 2 of the main manuscript that shows some of these features.

1. Histogram Analysis (**14 features**): The histogram contains the first-order statistical information about the image (or its fragment). Dividing the values histogram by the total number of pixels in the image one obtains the approximate probability density of occurrence of the intensity (greyscale) levels. Here, eight features were used to describe the texture
   1. Minimum: Minimum gray value of the pixels forming the region of interest
   2. Maximum: Maximum grayscale value of the pixels forming the region of interest
   3. Mean: Average grayscale value of the pixels forming the region of interest
   4. Median: Median grayscale value of the pixels forming the region of interest. More representative of the distribution, particularly if the distribution is biased.
   5. Variance: Variance is defined as the expectation of the squared deviation of a random variable from its mean. It is a measure of spread of the sample values from its mean
   6. Standard deviation (SD): In statistics, the standard deviation is the usual way of measuring distance from the mean or median (it measures dispersion or variance)

$SD=\sqrt{\frac{\sum_{i=1}^{M} \sum_{j=1}^{N} (P\left(i,j\right)-Mean)^{2}}{MN}}$, where P(i, j) is a grayscale value at a pixel at location row i and column j of an image of size M x N (row x column)

- 1. Quartile Range (QR): Where a range is a measure of where the beginning and end are in a distribution**, an interquartile range is a measure of where the middle 50% of the distribution lie.**

$QR=\text{ }\mathbf{Q}_{\mathbf{3}} -\text{ }\mathbf{Q}_{\mathbf{1}}.$**where Q3 and Q1 are 3^rd^ and 1^st^ quartile respectively**

- 1. Percentile (5 features): Percent of pixels above 2.5%, 25%, 50%,75% and 97.5%
  2. Skewness (SKEW): Skewness is a measure of the lack of symmetry in a distribution. A symmetrical distribution has a skew of zero. A positive skewness value indicates a positively skewed distribution and likewise a negative skewness value indicates a negatively skewed distribution.

- 1. Kurtosis (KURT): It is the fourth moment in statistics. Kurtosis is indicative of the “peakedness’ of the distribution. Technically, a measure of close the values of the distribution are to the mean. A positive value indicates less outliers and more peaked, likewise a negative value indicates too many outliers and less peaked.

$$KURT=\frac{\sum_{i=1}^{M} \sum_{j=1}^{N} \left[\frac{(P\left(i,j\right)-MEAN)}{SD}\right]^{4}-3}{MN}$$

Histogram analysis is completely based on the distribution of the grayscale values forming the region of interest; it provides no information about the spatial relationship of the pixels to each other. Therefore, differentiating 2 completely different texture patterns with the same number of black and white pixel but different orientation is not possible.

2. Two-dimensional and Three-dimensional Gray-level co-occurrence method (GLCM) and Gray-level difference method (GLDM) Analysis (**19 features each**): Secondly, we performed second order statistical analysis of texture, which included 2D- GLCM and GLDM analysis. These analyses took into account the both pixel intensities and their inter-relationships, thereby providing spatial information of the intensities (2nd order texture analysis) in various forms. For workflow implementation, the number of gray levels were reduced to 12-bit, which was determined to be sufficiently accurate for the study of texture. 19 different features were calculated, 13 based on the method by Haralick^3,4^ and seven additional features. The 19 features used to describe the texture within the region of interest include

1. Angular second moment (ASM):

$$ASM=\sum_{i} \sum_{j} \{P\left(i,j\right)\}^{2}$$

ASM reaches its highest value when grey level distribution are constant or repetitive, indicative of a homogenous distribution.

1. Uniformity: Uniformity is defined as the square root of ASM. Therefore, as in the case of ASM, uniformity reaches its highest value when grey level distribution are constant or repetitive, indicative of a homogenous distribution.
2. Contrast (CON):

$$CON=\sum_{i} \sum_{j} P\left(i,j\right)\left(i-j\right)^{2}$$

Also called sum of squares variance, CON weights pixels in the GLCM/GLDM map exponentially more as their distance from the diagonal increases. A larger value indicates greater variations in graylevels compared to their neighborhood.

1. Dissimilarity (DIS): Dissimilarity is similar to contrast, except that the weighting scheme is linear compared to exponential.
2. Homogeneity (HOM):

$$HOM=\sum_{i,j=0}^{N-1} \frac{P_{i,j}}{1+\left(i-j\right)^{2}}$$

HOM weights pixels in the GLCM/GLDM map exponentially less as their distance from the diagonal increases. A larger value indicates smaller variations in graylevels compared to their neighborhood. This is an inverse feature of CON.

1. Inverse Difference Moment (IDM): IDM is similar to HOM, except that the weighting scheme is linear compared to exponential.

$$IDM=\sum_{i,j=0}^{N-1} \frac{P_{i,j}}{1+\left(i-j\right)^{2}}$$

1. Inverse Difference Moment Normalized (IDMN): IDMN is similar to IDM, except that the weighting scheme is exponential and it is a measure of the local homogeneity of an image

1. Entropy (ENT):

$$ENT=-\sum_{i=1}^{M} \sum_{j=1}^{N} P\left(i,j\right)\log\left(P\left(i,j\right)\right)$$

ENT is a measure of randomness and have a higher value when the distribution is random, as opposed to orderly.

1. Correlation (CORR):

$$CORR=\sum_{i=1}^{M} \sum_{j=1}^{N} \left[\frac{((i,j)P\left(i,j\right)-MEAN_{ROW}MEAN_{COL})}{SD_{ROW}SD_{COL}}\right]$$

CORR measures the linear dependency of grey levels on those of neighboring pixels. Technically, it has a value of if uncorrelated, 1 if perfectly correlated.

1. Information measure of correlation 1(IMC1): IMC1 is based on the calculations of entropy values of Px and Py. For an uniform image (i.e., no pixel changes in that image), the IMC1 is zero. It produces negative values.

$$IMC1=\frac{-\sum_{i=1}^{M} \sum_{j=1}^{N} P\left(i,j\right)\log_{2}\left(P\left(i,j\right)\right)+\sum_{i=1}^{M} \sum_{j=1}^{N} P\left(i,j\right)\log_{2}\left(P_{x}\left(i\right),P_{y}\left(j\right)\right)}{MAX\left\{ENT\left(P_{x}\right),ENT\left(P_{y}\right)\right\}}$$

1. Information measure of correlation 2 (IMC2):

$$IMC2=\left\{1-\exp\left[-2\left(\sum_{i=0}^{N-1} \sum_{j=0}^{N-1} P_{x}\left(i\right)P_{y}\left(j\right)\log\left(P_{x}\left(i\right)\right)P_{y}\left(j\right)+\sum_{i=0}^{N-1} \sum_{j=0}^{N-1} P_{ij}\log_{2}P_{ij}\right)\right]\right\}^{1/2}$$

For an uniform image (i.e., no pixel changes in that image), the IMC2 is zero. It produces

values in the range of 0 to 1.

1. Sum of average (SUMAVER): For an image of single color of no variation, the sum average values for different angles are 2. Usually, for an image of varied pixel values, the sum average is high value.

$$SUMAVER=\sum_{i=0}^{2N-2} \left(i\right)\left(P_{x+y}\left(i\right)\right)where\left(P_{x+y}\left(k\right)\right)=\sum_{i=0}^{N-1} \sum_{j=0}^{N-1} P\left(i,j\right)$$

1. Sum of entropy (SUMENT): As per the definition of entropy (as mentioned above for F3), the value goes higher for an image of more variations.

$$SUMENT=\sum_{i=0}^{2N-2} P_{x+y}\left(i\right)\log\left(P_{x+y}\left(i\right)\right)$$

1. Sum of variance (SUMVAR): Usually, for an image of varied pixel values, the sum average is high value.

$$SUMVAR=\sum_{i=0}^{2N-2} P_{x+y}\left(i\right)\left(i-SUMAVER\right)^{2}$$

1. Difference of average and difference of entropy is calculated similar to sum of average and entropy respectively, except, differences are calculated instead of summations.
2. Standard deviation and mean are same as those calculated for histogram analysis, except run on GLCM / GLDM maps
3. Maximum correlation coefficient (MCC):

MCC= square root of second largest eigen value of Q, where$Q\left(i,j\right)=\sum_{k} \frac{g\left(i,k\right)g\left(j,k\right)}{g_{x}\left(i\right)g_{y}\left(k\right)}$

1. Maximum probability= max(p(i,j)); This feature captures the occurrences of the most dominant pair of neighboring intensity values.
2. Root mean square (RMS): RMS computes the root mean square value of each row or column of the input, along vectors of a specified dimension of the input, or of the entire input

$$RMS=\sqrt{\frac{\sum_{i=1}^{M} MEAN\left(i,j\right)^{2}}{M}}$$

3. ***FFT Analysis (3 features):*** FFT analysis was performed as previously described^5^. Specifically, a 512 point FFT was applied to all tumor images (ADC and T2). Using the in-built Matlab implementation of the FFT algorithm (FFT2), we extracted the individual frequencies, its amplitude (how much frequency of a given type is present) and phase (where in the image the frequency is present), of the original image. The resultant magnitude and phase of the FFT across all images were analyzed. Three features were defined. In all cases, the harmonics analysis was limited between 15% and 95% of maximum spatial frequency within the tumor. These cutoffs were chosen to avoid inclusion of low frequency content i.e., tumor size effect and noise, and the high frequency noise. The selected band-pass frequencies correspond to the spatial frequencies within the tumor.

1. Entropy of FFT mag (E_FFT_Mag): Diversity (Randomness) measure in the magnitude of FFT harmonics
   - $\boldsymbol{E\_FFT\_Mag}\boldsymbol{=-}\sum_{\boldsymbol{k=0}}^{\boldsymbol{n}} \boldsymbol{P}_{\boldsymbol{k}} \boldsymbol{Log}_{\boldsymbol{2}}\boldsymbol{P}_{\boldsymbol{k}}$**,** where $P_{k}$ is each harmonic from the FFT transformed (magnitude) tumor image.

*The E_FFT_Mag of a homogenous texture should be a smaller compared to the E_FFT_Mag of a heterogeneous texture.*

1. Entropy of FFT phase (E_FFT_Phase): Diversity (Randomness) measure in the phase of FFT harmonics
   - $\boldsymbol{E\_FFT\_Phase}\boldsymbol{=-}\sum_{\boldsymbol{k=0}}^{\boldsymbol{n}} \boldsymbol{P}_{\boldsymbol{k}} \boldsymbol{Log}_{\boldsymbol{2}}\boldsymbol{P}_{\boldsymbol{k}}$, where $P_{k}$ is every harmonic from the FFT transformed (phase) tumor image.

*The E_FFT_Phase of a homogenous texture should be a smaller compared to the E_FFT_Phase of a heterogeneous texture.*

1. Complexity index (CI): Sum of the amplitude of all FFT harmonics
   - $\boldsymbol{CI}\boldsymbol{=}\sum_{\boldsymbol{k=0}}^{\boldsymbol{n}} \boldsymbol{P}_{\boldsymbol{k}}$, where $P_{k}$ is every harmonic from the FFT transformed (amplitude) tumor image.

*The CI of a homogenous texture should be a smaller value compared to the CI of a heterogeneous texture.*

**References**

1. Cameron A, Khalvati F, Haider MA, Wong A. MAPS: a quantitative radiomics approach for prostate cancer detection. IEEE Transactions on Biomedical Engineering. 2016 Jun;63(6):1145-56.

2. Aggarwal N, Agrawal RK. First and second order statistics features for classification of magnetic resonance brain images. Journal of Signal and Information Processing. 2012 May 30;3(02):146.

3. Larroza A, Bodí V, Moratal D. Texture analysis in magnetic resonance imaging: review and considerations for future applications. InAssessment of Cellular and Organ Function and Dysfunction using Direct and Derived MRI Methodologies 2016. InTech.

4. Haralick RM, Shanmugam K. Textural features for image classification. IEEE Transactions on systems, man, and cybernetics. 1973 Nov(6):610-21.

5. Varghese BA, Hwang DH, Cen SY, Desai BB, Yap FY, Gill I, Desai M, Aron M, Liang G, Chang M, Deng C. Fast Fourier transform-based analysis of renal masses on contrast-enhanced computed tomography images for grading of tumor. In 12th International Symposium on Medical Information Processing and Analysis 2017 Jan 26 (Vol. 10160, p. 101600J). International Society for Optics and Photonics.
